# Medical complexity and healthcare utilization among patients attending three U.S. post-COVID clinics

**DOI:** 10.1186/s12879-025-11424-1

**Published:** 2025-09-26

**Authors:** Miriam A. M. Nji, Elizabeth M. Briones, Anindita Issa, Maureen Tierney, Jeanne Bertolli, Surendra Barshikar, Elizabeth R. Unger, Juan Wisnivesky, Quan Vu, David Quimby, Joseph Abrams, Nikhil Jagan, Sasha Manouchehripour, Martin Laguerre, Jennifer R. Cope

**Affiliations:** 1 National Center for Emerging and Zoonotic Infections, Centers for Disease Control and Prevention, Atlanta, GA USA; 2 Creighton University School of Medicine, CHI Health, Omaha, NE USA; 3 University of Texas Southwestern Medical Center, Dallas, TX USA; 4 Division of General Internal Medicine, Icahn School of Medicine at Mount Sinai, New York City, NY USA

**Keywords:** post-COVID conditions, post-COVID clinics, SARS-CoV-2, COVID-19 sequelae, Long COVID, Post-acute sequelae of SARS-CoV-2

## Abstract

**Background:**

Patients who do not fully recover or develop new symptoms following SARS-CoV-2 infection require follow-up and sometimes seek care at specialized multidisciplinary care clinics. We aimed to describe the clinical characteristics and care needs of patients at three such post-COVID clinics.

**Methods:**

We conducted a multisite retrospective electronic chart review of 984 patients, aged ≥ 18 years, who visited one of three post-COVID clinics at least 28 days after a clinical or polymerase chain reaction (PCR)-confirmed diagnosis of SARS-CoV-2 infection between January 20, 2020, and March 31, 2021. The clinics were located in Omaha, Nebraska, New York City, New York, and Dallas, Texas. Patient records were obtained through September 30, 2021. Data on clinical evaluations and healthcare provider visits were abstracted by trained clinical personnel using a standardized health record abstraction tool.

**Results:**

The median age was 52 years (range 18–89 years), 59.9% were female, and 69.0% were White. Of 984 patients, 79.9% had SARS-CoV-2 infection that was confirmed by PCR, 32.1% had three or more comorbid conditions, and 39.4% had been hospitalized. During post-COVID follow-up, the most common symptoms were shortness of breath (59.2%), post-exertional malaise (45.6%), fatigue (43.2%), and brain fog (42.8%). Nearly one in three patients had a diagnosis of post-viral fatigue syndrome (30.1%), and pulmonary system conditions (24.4%) were also common. Overall, the 984 participants attended 3914 visits (median 3; range 1–46) over a median follow-up period of 107 days (range 1–560) between first and last post-COVID follow-up visits. Of the 984 patients, 64.3% were referred for subspecialty care notably pulmonology, cardiology, and neurology. More than a third of patients were referred for rehabilitation therapy (37.9%) including physical, occupational, speech, and psychotherapy.

**Conclusion:**

Adult patients at post-COVID clinics have a wide range of symptoms and conditions that highlight the medical complexity of these patients and their need for high levels of care, including multiple health care visits and referrals for therapy. This underscores the need for well-coordinated, multidisciplinary care, and planning of health resources for post-COVID-19 follow-up care.

**Supplementary Information:**

The online version contains supplementary material available at 10.1186/s12879-025-11424-1.

## Background

Through March 2023, over 100 million people in the United States had been infected with severe acute respiratory syndrome coronavirus 2 (SARS-CoV-2), the causative agent of COVID-19 [1]. While most people recover fully from the infection, it is evident that some have a spectrum of new onset, worsening, or persistent symptoms, known as post-COVID-19 conditions (PCC), Long COVID, or post-acute sequelae of SARS-CoV-2 (PASC) [2–5]. People who were hospitalized for SARS-CoV-2 infection were at higher risk of persistent post-acute symptoms [6–8]. However, most people with PCC had a mild acute SARS-CoV-2 infection [9]. As estimated from the Household Pulse Survey conducted by the U.S. Census Bureau in July 2023, 15.4% of U.S. adults who ever had COVID-19 currently also had symptoms consistent with Long COVID, up to 25% of whom reported significant activity limitations from these symptoms [10–11]. Other studies have reported that persistent symptoms after SARS-CoV-2 infection can affect patients’ ability to think and concentrate (commonly referred to as brain fog), their ability to work or participate in physical activity, their social interactions with others, and their overall quality of life [8, 12–14]. Patients who experience PCC may have symptoms for a year or more after their initial COVID-19 infection [8, 15–16].

Because of the breadth of reported symptoms, multi-organ involvement, heterogeneity, and complexity of PCC, multidisciplinary clinics emerged across the United States to provide care for patients with PCC. By December 2022, almost every state had at least one clinic specializing in post-COVID care [17], with a total of 66 interdisciplinary clinics, according to Becker’s Hospital Review in 2022 [18]. As these clinics emerged, research that could potentially guide post-COVID care was making progress. Lacking a standard definition and consensus on how to diagnose patients, post-COVID clinics began setting standards for post-COVID care. However, data on which to base clinical care continues to be limited [19]. Characterizing the patients and care patterns in these clinics could help guide identification of PCC and improve delivery of and access to care in other settings. Therefore, we aimed to describe the clinical characteristics of patients who attended post-COVID clinics, with a focus on medical complexity and their care patterns during post-COVID follow-up.

## Methods

This was a multisite, retrospective clinical chart review of a cohort of patients who attended one of the three post-COVID clinics. The post-COVID clinics were at three university hospital centers: Mount Sinai Health System (MSHS), New York City, New York; University of Texas Southwestern Medical Center (UTSW), Dallas, Texas; and CHI Health Creighton University Medical Center (CHI), Omaha, Nebraska. The selected centers were among the first to establish specialized clinics for post-acute COVID-19 care and had seen a significant number of patients. All three clinics operate slightly differently, but they each use a multidisciplinary structured approach to assess and develop individualized care for patients. The clinical care teams were led by a pulmonologist, an endocrinologist, and/or a physiatrist, alongside other experts in primary care, psychiatry, social work, and other health care specialties. Patients were referred by the hospital acute care teams, community-based clinical providers, or self-referred. The frequency and number of visits was informed by clinical evaluation and involved shared decision making between the clinicians and patients. Clinical assessments were tailored to the patient’s presentation, and when indicated, patients were referred for subspecialty care.

Patient charts were randomly selected from the cohort of patients at each site. To reach a goal of 1,000 patients for this study, we aimed to select approximately 1,100, to allow for exclusion of patients not meeting eligibility criteria. Patients included were 18 years or older,

- had either a positive SARS-CoV-2 PCR or a COVID-19 clinical diagnosis between January 20, 2020, and March 31, 2021, and,
- had at least one clinical encounter with a healthcare provider ≥ 28 days from the date of symptom onset or date of PCR diagnosis, whichever came first (for non-hospitalized patients) or ≥ 28 days from the date of first discharge (for patients hospitalized for acute infection).

We excluded patients who were missing key demographic variables like age and sex. A standardized health record abstraction tool, designed in the Research Electronic Data Capture (REDCap) software, was piloted and used for data collection. Medical records were reviewed, and data were abstracted by trained clinical personnel. Data obtained included information on demographics, pre-existing medical conditions, acute COVID-19 illness course and management, and all post-acute COVID-19 follow-up visits. Specifically, for the follow-up visits, we collected data on patients’ presenting symptoms, findings from clinical assessments, laboratory and imaging results for all visits that met eligibility criteria and occurred through September 30, 2021. To ensure data quality, 42 key variables were re-abstracted by a second reviewer for 5% of all charts. Discrepancies in abstracted data were addressed via consensus, including a third reviewer when necessary. Inter-rater reliability was measured, and the average percentage of agreement between primary and secondary abstractors was 88%.

This activity was reviewed by CDC and was determined to not meet the definition of research as defined in 45 Code of Federal Regulations 46.102(l) as it does not develop or contribute to generalizable knowledge. It was conducted consistent with applicable federal law and CDC policy. After reviewing the activity, the Institutional Review Boards at the three sites waived informed consent and approved the activity.

Outcomes of interest were clinical characteristics of adult patients and medical care patterns during post-COVID follow-up. Clinical characteristics following acute SARS-CoV-2 infection included any symptoms, results of physical examination, objective findings, and diagnoses occurring at least 28 days after the index date. The index date was the date of first PCR or clinical diagnosis of SARS-CoV-2 for non-hospitalized patients or date of discharge for hospitalized patients. Care patterns were described based on visit characteristics including the number of visits, interaction model (in-person or telehealth), and care setting (primary care, subspecialist, emergency department, or specialized clinic for PCC).

Other data collected were demographic characteristics (sex, age, race/ethnicity, health insurance, and employment status), comorbid conditions, and data on the acute phase of SARS-CoV-2 infection. Information collected on the disease course included: diagnosis date, type of diagnosis (PCR-confirmed vs. clinical), date of symptom onset, symptoms present, and care setting (inpatient or ambulatory care). If the patient was hospitalized, we gathered information on hospitalization and discharge dates, as well as the level of specialized care.

Post-COVID follow-up data collected at each eligible visit included information on therapy-focused visits (type and frequency) and other follow-up visits (date of visit, need for sub-specialty care, chief complaints, symptoms present, objective physical exam findings, standardized screening, laboratory tests, imaging, and diagnoses).

The Charlson comorbidity index was calculated using the weighted indices of comorbidities, including those commonly seen in PCC such as diabetes, as well as decade of age for those 50 years or more [20–22].

SAS version 9.4 (SAS Institute, Cary NC) was used for the analyses. Data are presented as frequencies and percentages for categorical variables and medians (range) for continuous variables.

## Results

A total of 1051 patients were identified through a simple random sample. After exclusion of 67 ineligible patients, data abstracted from the medical records of 984 patients were used in this analysis (UTSW *n* = 326; CHI *n* = 308; MSHS *n* = 350).

The median age of patients was 52 years (range 18–89 years), 59.9% were female, and 69.0% were White. A majority (79.9%) of patients had PCR-confirmed SARS-CoV-2 infection (Table 1).

**Table 1:** Characteristics of adult patients attending three Post-COVID Clinics—United States, January 20, 2020–March 31, 2021

Variable	Total (*n* = 984)
Age, median (range) years	52 (18−89)
Female, n (%)	589 (59.9)
Race, n (%)	
White	678 (69.0)
Black	88 (9.0)
Asian	18 (1.8)
Native American	6 (0.6)
Pacific Islander	2 (0.2)
Other/multiracial	49 (5.0)
Unknown	142 (14.5)
Pre-existing conditions (comorbidities), n (%)	
Hypertension	346 (35.2)
Obesity (BMI > 30)	313 (31.9)
Smoking history, current and former	309 (31.4)
Sleep disorders	160 (16.0)
Asthma	156 (15.9)
Diabetes	152 (15.5)
Generalized anxiety disorder	141 (14.3)
Major depressive disorder	135 (13.7)
Cancers	72 (7.3)
Coronary artery disease	65 (6.6)
Chronic obstructive pulmonary disease	58 (5.9)
Chronic kidney disease	40 (4.1)
Heart failure	34 (3.5)
Stroke or transient ischemic attacks	26 (2.6)
Myocardial infarction	13 (1.3)
Liver disease	13 (1.3)
Post-traumatic stress disorder	12 (1.2)
Cardiomyopathy	8 (0.8)
Peptic ulcer disease	3 (0.3)
Dementia	3 (0.3)
Organ transplant	2 (0.1)
Comorbidity total, n (%)	
0	255 (25.9)
1–2	413 (42.0)
3 or more	316 (32.1)
Charlson Comorbidity Index^a^, median (range)	1 (0–11)
Diagnosis of SARS-CoV-2 infection, n (%)	
PCR-confirmed	783 (79.9)
Clinical presumption	197 (20.1)
Care status during acute infection^b^, n (%)	
Hospitalized	388 (39.4)
Outpatient, Emergency room	371 (37.8)
Outpatient, Other ambulatory services	280 (28.5)

Mount Sinai Health System (MSHS), New York City, New York; University of Texas Southwestern Medical Center (UTSW), Dallas, Texas; and CHI Health Creighton University Medical Center (CHI), Omaha, Nebraska

The index date is date of PCR or clinical diagnosis for non-hospitalized patients and date of discharge for hospitalized patients during the acute illness phase of SARS-CoV-2 infection. Diagnosis of SARS-CoV-2-infection occurred between January 20, 2020, to March 31, 2021.

*PCR* polymerase chain reaction, *COPD* chronic obstructive pulmonary disease, BMI body mass index, *SARS-COV-2* severe acute respiratory syndrome coronavirus 2

^a^ The Charlson comorbidity index is a weighted score that predicts the 10-year mortality of patients based of age and comorbidities [21, 22]. The lowest score of 0 corresponds to a 98% estimated survival rate.

^b^ Categories are not mutually exclusive. Some patients received care during the acute phase in more than one setting.

During the acute phase of SARS-CoV-2 infection, 651 (66.3%) received care in outpatient settings, including emergency room visits and other ambulatory services, and 388 (39.4%) patients were hospitalized (Table 1). During the acute illness, patients who had symptoms presented with at least one of the following: fever or chills (63.8%), cough (64.1%), shortness of breath (66.0%), fatigue (68.8%), and muscle/body aches (53.9%) (not tabulated). A small proportion (2.5%) of patients were asymptomatic during their acute illness.

The most frequently recorded pre-existing conditions were hypertension (35.2%), obesity (31.9%), and history of ever smoking (31.4%). About three in four patients had at least one comorbid condition, and 32.1% had three or more comorbid conditions. The median Charlson Comorbidity Index was 1 (range = 0–11) (Table 1).

The most common presenting symptoms at post-COVID follow-up visits were shortness of breath (59.2%), post-exertional malaise (45.6%), fatigue (43.2%), and brain fog (42.8%) (Supplemental Table 1). Thirty-two patients had virtual evaluations via telehealth visits and therefore did not have a physical exam assessment. When physical exams were performed (*n* = 952), more than half the patients (57.4%) had no abnormal findings on physical exam at any of their visits. This includes no abnormal vital signs, hypoxemia, or other abnormalities on routine exam. The most common abnormalities seen in the 42.6% patients who did have at least one abnormality were hypoxemia, need for supplemental oxygen at time of visit, sensory loss, rash, hearing loss, and abnormal mental status (Supplemental Table 1).

**Fig. 1:**
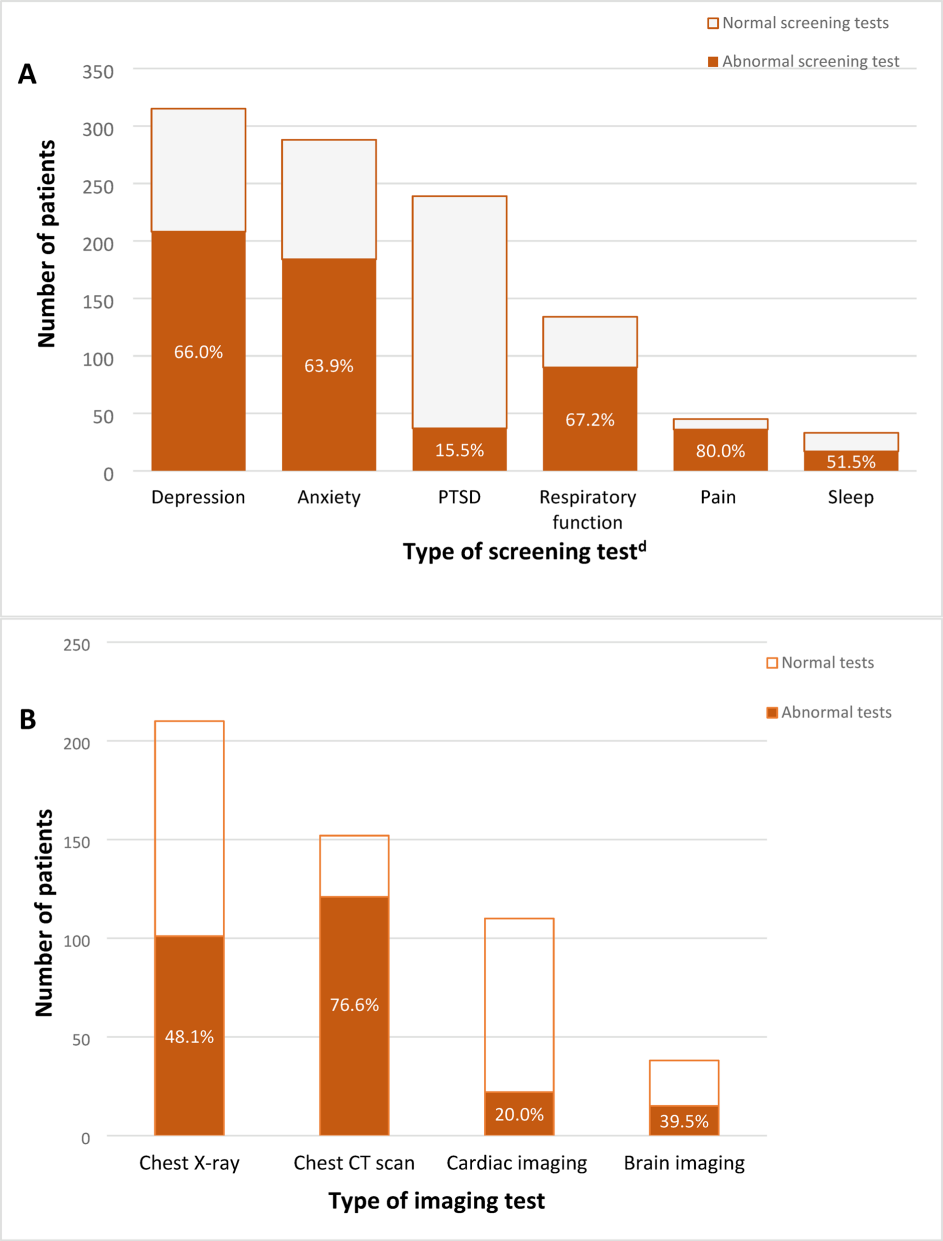
Yield of Abnormalities Detected in Screening^a^ (**A**) and Imaging Tests^a^ (**B**) Conducted During Health Care Visits for Adults at Three Post-COVID Clinics—United States^b^, January 20, 2020–March 31, 2021^c^. *PTSD* post-traumatic stress disorder, *CT* computerized tomography, *MRI* magnetic resonance imaging. ^a^Screening and imaging tests were done based on clinical presentation and at the discretion of the provider. Overall, 468 patients were screened for at least one of the conditions, and 394 patients had at least one imaging test done. However, the total number of patients who were administered screening or imaging tests varied for each test and can be determined by the height of the bars. The percentages in the darker portion of the bars represents the percentage of tests that were abnormal for each test. ^b^Mount Sinai Health System (MSHS), New York City, New York; University of Texas Southwestern Medical Center (UTSW), Dallas, Texas; and CHI Health Creighton University Medical Center (CHI), Omaha, Nebraska. ^c^Visits occurred ≥ 28 days after the index date through September 30, 2021. The index date is date of PCR or clinical diagnosis for non-hospitalized and date of discharge for hospitalized patients during the acute illness phase of SARS-CoV-2 infection. Diagnosis of SARS-CoV-2-infection occurred between January 20, 2020, to March 31, 2021. ^d^Standardized screening tools used were the Patient Health Questionnaire forms (PHQ-2 and PHQ-9) for depression; General Anxiety Disorder-7 for anxiety; Impact of Event Scale-6, PTSD checklist for DSM5 (PCL5), and PTSD screener for PTSD; Montreal Cognitive Assessment, Mini-mental State Examination, and Mini-cog for cognitive impairment; pain numeric scale for pain; Modified Medical Research Council dyspnea scale and Borg scale for pulmonary symptoms; Epworth sleepiness scale and Stop Bang scale for sleep. Other screening tests used were 6-minute walk test, 10-minute lean test.

When indicated, standardized tests were used to screen for conditions, namely depression, anxiety, post-traumatic stress disorder (PTSD), respiratory function, pain, and sleep disorders. A total of 468 patients had at least one standardized screening test administered. Five of the six tests listed above revealed abnormal results in a majority of patients (range 51.5–80.0%). Only the PTSD screening test had a small percentage (15.5%) of patients with abnormal results (Fig. 1A). The imaging tests included chest x-ray, chest computed tomography (CT) scans, cardiac imaging, and brain imaging. A total of 394 patients had at least one imaging test performed, and the percentage of abnormal chest CT scans was highest at 76.6%. However, when ordered, chest X-ray, cardiac imaging, and brain imaging yielded lower proportions of abnormal findings (48.1%, 20.0%, and 39.5%, respectively) (Fig. 1B). Of patients with documented laboratory tests, abnormal results were most often returned for serum glucose (47.6%), serum albumin (31.3%), and estimated glomerular filtration rate (31.0%) (Supplemental Table 2).

**Fig. 2:**
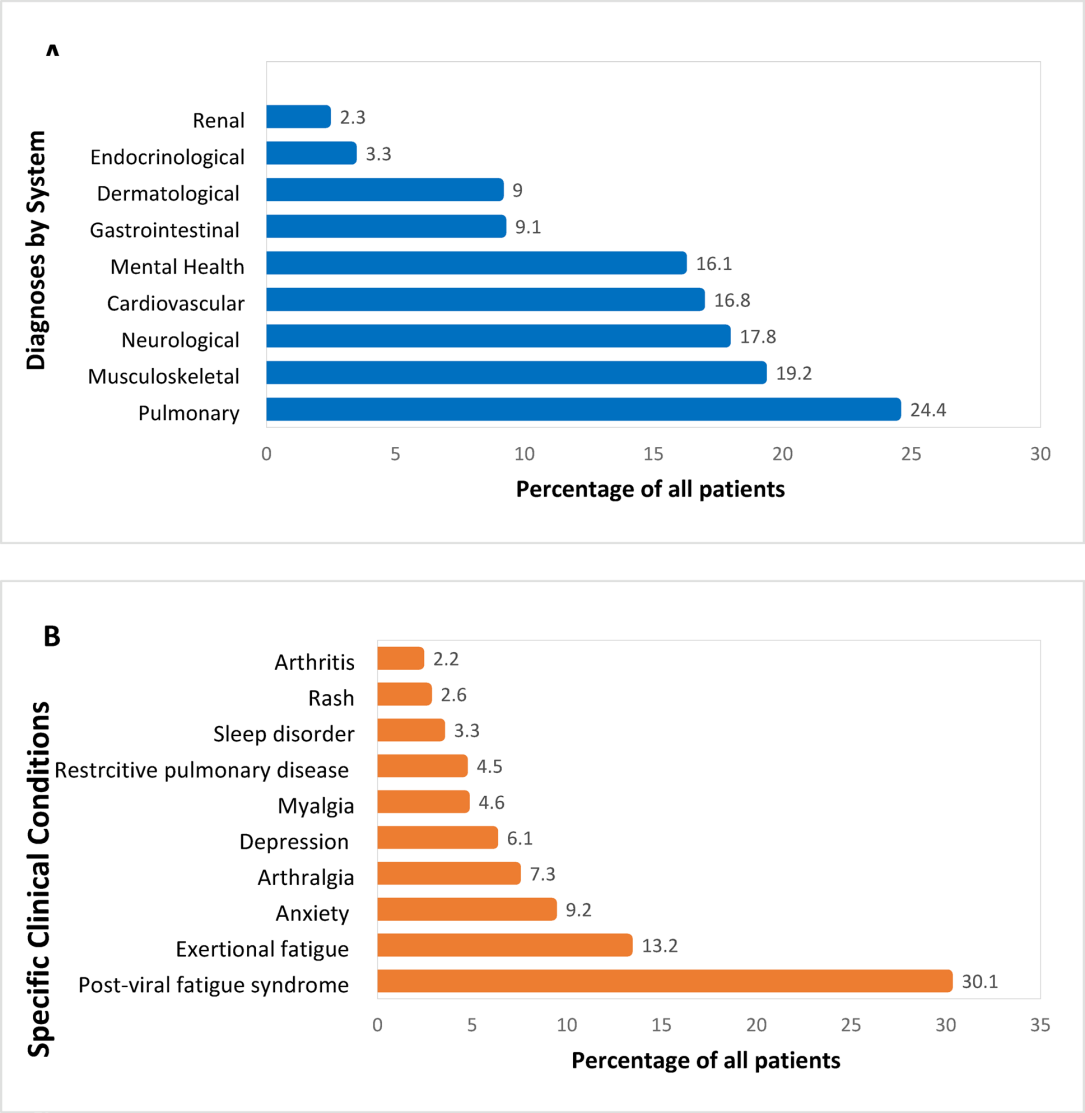
Percentage of Patients with Diagnoses by System (**A**) and Specific Clinical Conditions (**B**) at Health Care Visits of Adult Patients Attending Three Post-COVID Clinics—United States^a^, January 20, 2020–March 31, 2021^b^. ^a^ Mount Sinai Health System (MSHS), New York City, New York; University of Texas Southwestern Medical Center (UTSW), Dallas, Texas; and CHI Health Creighton University Medical Center (CHI), Omaha, Nebraska. ^b^ Visits occurred ≥ 28 days after the index date through September 30, 2021. The index date is date of PCR or clinical diagnosis for non-hospitalized and date of discharge for hospitalized patients during the acute illness phase of SARS-CoV-2 infection. Diagnosis of SARS-CoV-2-infection occurred between January 20, 2020, to March 31, 2021.

The most common diagnosis of a single condition was post-viral fatigue syndrome (30.1%). Pulmonary system conditions, such as pneumonia, fibrosis, restrictive pulmonary disease, pulmonary artery hypertension, pulmonary embolism, and deterioration of prior chronic pulmonary disease, were also common (24.4%) (Fig. 2A). (Fig. 2B).

Overall, the 984 patients attended a combined total of 3,914 visits (median 3; range 1–46). About a quarter of patients (24.1%) had a single PCC visit during the study follow-up period, 47.2% had 2–4 visits, 22.9% had 5–10 visits, and 5.9% had > 10 visits (Table 2). The median number of days from acute infection to the first post-COVID follow-up visit was 98 (range 28–572 days). The median length of time from first to last post-COVID follow-up visit was 107 days (range 1–560 days). To provide a more detailed picture of the intensity of care, we report follow-up parameters by the number of visits and length of follow-up (Table 2). All patients with only one visit had a follow-up period of ≤ 90 days. Patients with 5–10 or > 10 visits were typically followed for longer than 180 days. However, 36.1% (134/371) of patients who had a short length of follow-up (≤ 90 days) had multiple (> 1) visits. Of the 387 patients who were followed for more than 180 days, 89 (23.0%) had at least one visit per month.

**Table 2:** Summary of health care visits for adult patients at three Post-COVID Clinics—United States, January 20, 2020–March 31, 2021

Variables	Number of Patients by Post-COVID Visit Category *n*, (%)				
	Total (*n* = 984)	1 Visit (*n* = 237)	2–4 Visits (*n* = 464)	5–10 Visits (*n* = 225)	> 10 Visits (*n* = 58)
Length of follow up (days)					
28–90	371 (37.7)	237 (100.0)	131 (28.2)	3 (1.3)	0
91–180	226 (23.0)	0	175 (37.7)	46 (20.4)	5 (8.6)
181–270	202 (20.5)	0	102 (22.0)	80 (35.6)	20 (34.5)
271–365	101 (10.3)	0	33 (7.1)	51 (22.7)	17 (29.3)
> 365	84 (8.5)	0	23 (5.0)	45 (20.0)	16 (27.6)
Visit specialty^a^					
Post-COVID clinic (unspecified)^b^	737 (74.9)	166 (70.0)	361 (77.8)	165 (73.3)	45 (77.6)
Primary care	392 (39.8)	15 (6.3)	190 (40.9)	145 (64.4)	42 (72.4)
Sub-specialty	633 (64.3)	56 (23.6)	313 (67.5)	206 (91.6)	58 (100.0)
Pulmonary	246 (25.0)	37 (15.6)	95 (20.5)	88 (39.1)	26 (44.8)
Cardiology	220 (22.4)	1 (0.4)	97 (20.9)	91 (40.4)	31 (53.4)
Neurology	89 (9.0)	2 (0.8)	40 (8.6)	31 (13.8)	16 (27.6)
Ear-Nose-Throat	49 (5.0)	1 (0.4)	13 (2.8)	29 (12.9)	6 (10.3)
Rheumatology	41 (4.2)	1 (0.4)	12 (2.6)	20 (8.9)	8 (13.8)
GI/Hepatology	39 (4.0)	0	10 (2.2)	21 (9.3)	8 (13.8)
Physiatry	38 (3.9)	10 (4.2)	22 (4.7)	4 (1.8)	2 (3.4)
Dermatology	36 (3.7)	0	15 (3.2)	13 (5.8)	8 (13.8)
Obstetrics/Gynecology	32 (3.3)	0	9 (1.9)	16 (7.1)	7 (12.1)
Hematology/Oncology	22 (2.2)	0	7 (1.5)	9 (4.0)	6 (10.3)
Endocrinology	21 (2.1)	0	4 (0.9)	10 (4.4)	7 (12.1)
Nephrology	12 (1.2)	0	2 (0.4)	6 (2.7)	4 (6.9)
Ophthalmology	11 (1.1)	0	1 (0.2)	7 (3.1)	3 (5.2)
Infectious diseases	8 (0.8)	0	2 (0.4)	5 (2.2)	1 (1.7)
Therapy^c^	373 (37.9)	54 (22.8)	195 (42.0)	93 (41.3)	31 (53.4)
Physical therapy	287 (29.2)	40 (16.9)	152 (32.8)	71 (31.6)	24 (41.4)
Speech therapy	101 (10.3)	12 (5.1)	59 (12.7)	21 (9.3)	9 (15.5)
Psychotherapy	67 (6.8)	7 (3.0)	39 (8.4)	14 (5.5)	7 (12.1)
Occupational therapy	45 (4.6)	5 (2.1)	23 (5.0)	10 (4.4)	7 (12.1)
Post-COVID hospital admissions^d^	88 (8.9)	6 (2.5)	36 (7.8)	29 (12.9)	17 (29.3)

Mount Sinai Health System (MSHS), New York City, New York; University of Texas Southwestern Medical Center (UTSW), Dallas, Texas; and CHI Health Creighton University Medical Center (CHI), Omaha, Nebraska

Visits occurred ≥ 28 days after the index date through September 30, 2021. The index date is date of PCR or clinical diagnosis for non-hospitalized patients and date of discharge for hospitalized patients during the acute illness phase of SARS-CoV-2 infection. Diagnosis of SARS-CoV-2-infection occurred between January 20, 2020, to March 31, 2021.

*GI* gastrointestinal, *PT/OT* physical therapy/ occupational therapy

^a^ Visit specialty groups are not mutually exclusive

^b^ Interdisciplinary care at post-COVID clinics represent visits during which the patient saw multiple (2 or more) specialties at the same visit.

^c^ Type of therapy categories are not mutually exclusive. Overall, 373 patients were prescribed therapy at any point during the follow-up. Other types of therapy included nutrition therapy (*n* = 1), olfactory therapy (*n* = 2), aromatherapy (*n* = 2) music therapy (*n* = 3), post-COVID group therapy (*n* = 6), cardiopulmonary therapy (*n* = 19), unspecified (*n* = 31).

^d^ Hospital admissions included instances of hospitalization and emergency room visits during the follow-up period.

During the follow-up period, 74.9% visited the PCC clinic with an unspecified provider type, 64.3% visited subspecialty care, and 39.8% of patients visited their primary care providers. The most common subspecialty visits were pulmonology (25.0%), cardiology (22.4%), and neurology (9.0%) services. As the number of visits increased, so did the proportion of referrals for subspecialty care.,The following percentages of patients visited subspecialty care: 23.6% of those with one visit of any type; 67.5% of those with 2–4 visits; 91.6% of those with 5–10 visits, and 100% of those with > 10 visits. For patients who had > 10 visits (*n* = 58), 72.4% were seen by a provider within one year of their COVID diagnosis. Of this subset of patients with > 10 visits, 72.4% visited a primary care provider at least once. Of all patients, 88 (8.9%) were hospitalized at least once during the follow-up period (Table 2).

Of the 984 patients, 373 (37.9%) of them had at least one rehabilitation therapy-related visit. Most patients had visits for physical therapy (29.2%), speech therapy (10.3%), and/or psychotherapy (6.8%). Only 4.6% of patients had occupational therapy visits, and 1.9% had cardiopulmonary therapy. Other forms of therapy, such as nutrition therapy, music therapy, aromatherapy, olfactory and/or post-COVID wellness group therapy, were also prescribed for 12.1% of patients. Across all types of rehabilitation therapy, the proportion of patients who were prescribed therapy was higher among patients with at least 5 visits of any type (43.8%) compared to patients with one to four visits (35.5%) (Table 2).

## Discussion

This review of patient records from three multidisciplinary post-COVID clinics documents the medical complexity of post-COVID conditions and the need for coordinated care. Three-quarters of the patients with PCC in this study had at least one comorbidity (most commonly hypertension, obesity, and smoking history), and this finding aligns with reports of high percentages of comorbidities among patients with acute SARS-CoV-2 infection [23]. In this middle-aged cohort with predominantly mild to moderate acute COVID-19 illness, patients attended multiple post-COVID visits (median 3, range 1–46) with some patients coming in more than once a month over extensive periods of follow-up, comparable to prior reports for patients with PCC and other chronic conditions [24–26].

The concept of complexity in adult patients lacks consensus among healthcare providers and researchers, but generally involves the interrelationship between many medical and non-medical factors [27]. Patients with post-COVID conditions in this study can be characterized as medically complex because of their pre-existing comorbidities, acute illness severity, and post-acute multiorgan involvement. Other medical factors that contribute to their complexity include the need for numerous visits requiring in-depth assessments over a prolonged period, as well as services of subspecialists and rehabilitation therapists [27, 28].

In the post-COVID phase, most patients in our study experienced a range of symptoms (most commonly dyspnea, post-exertional malaise, and fatigue) involving multiple organ systems. Results from mental health questionnaires suggested possible mental health conditions needing additional follow-up in over half of patients in this study. PCC has been associated with poor quality of life and limitations in activities of daily living, as well as mental health conditions [12, 13]. Certain conditions associated with COVID-19 have been reported to worsen over time, months after initial illness [25]. This could explain the longer lengths of follow-up and multiple visits among some patients.

Patients in this study required comprehensive clinical evaluations. To determine potential causes of symptoms and physical exam findings (or lack thereof in many cases), some patients required further testing such as screening tests, blood tests, and imaging studies. These yielded abnormal results in most patients on chest computed tomography, pulmonary function tests, and screening questionnaires (for mental health, pain, and sleep). However, the results of some tests, like cardiac imaging, brain imaging, and the majority of blood tests, were normal in most patients. While not explicitly stated in the chart, clinicians presumably requested these tests, which are sometimes burdensome in terms of time and cost to patients, to guide differential diagnosis and exclude other treatable conditions. In line with clinical findings, pulmonary system diagnoses were common. However, the most common diagnosis was post-viral fatigue syndrome (30.1%), a complex debilitating condition that requires investigation of numerous other potential causes of fatigue.

Patients had multiple provider visits in primary care, as well as subspecialist care and referrals for rehabilitation therapy. Generally, as the number of visits increased, so did the length of follow-up and the number of subspecialists and therapists. More than two-thirds of patients had at least one subspecialty visit during their follow-up. Subspecialists were primarily pulmonologists, neurologists, and cardiologists, which reflects the care needs outlined by providers at other post-COVID clinics (29. Without evidence-based therapies, multiple visits to a range of specialists might be required to determine how best to manage symptoms associated with PCC [29]. While primary care played a role in the management of these patients, these high rates of subspecialty referrals might signal challenges in care coordination that have been reported in other studies, such as increased wait times and frustration among patients [30].

Nearly 40% of patients required physical therapy, occupational therapy, speech therapy or psychotherapy. The significant proportion of post-COVID patients requiring these types of prolonged care indicates healthcare systems and individual patients might experience financial and other resource burdens. The amount of time and effort patients need to commit to therapy sessions may interfere with their work schedules, thus making it more challenging to return to pre-illness work productivity. Despite the financial and time commitments for therapy sessions, the benefits of attending therapy and recovering pre-illness function as efficiently and safely as possible have been demonstrated in other studies [31]. One study suggested that patients with PCC may have poorer physical/functional health and were significantly more likely to use health care services than their counterparts in cancer rehabilitation [24].

### Strengths and limitations

Among the strengths of this study was that it investigated medical care early in the recognition of PCC, and therefore, could potentially serve as a benchmark for how care has evolved since then. Another strength is that the specialized services and collaborative management plans were at the time unique to university health systems, like those in this study. This structure contributed to the quality of clinical description and documentation of medical care for this investigation.

The results of this study should be interpreted in the context of some limitations. First, our study may be subject to selection bias because of the nonrandom selection of the three clinics (they were among the first post-COVID clinics in the country with substantial numbers of patients in attendance and had the capacity to conduct research). These findings may not represent the profile of patients at other clinics in smaller communities or rural settings. By design, we recruited only patients who had a healthcare encounter at a clinic. Thus, we did not include all the other patients who did not attend clinic for various reasons, such as not having health insurance, residing too far from the clinics (which were predominantly in large urban areas), and not seeking care for milder post-COVID symptoms. Participants in our study may not reflect the sociodemographic distribution of those affected by PCC in the catchment areas of the participating clinics. Therefore, our results may not be generalizable. We describe the complexity of patients and their care. However, we cannot be certain that all the visits and tests were directly related to their previous SARS-CoV-2 infection as we lack a control group for comparison. Other studies have reported a significant increase in healthcare use directly attributable to COVID-19 sequelae [20, 21]. Since our study recruited from a post-COVID clinic, it could be assumed that patients came in for COVID-related care. Second, it is possible that we underestimated the number of visits, as out-of-system provider visits were not captured in our data collection tool. Lastly, 20% of our patients had clinically diagnosed SARS-CoV-2 infection without a confirmatory test and could have had symptoms from other causes misclassified as PCC.

## Conclusion

Patients at post-COVID clinics have a wide range of symptoms and conditions that highlight their complex medical profiles and need for high levels of care, including multiple healthcare visits. This study has particularly underscored the need for subspecialists and rehabilitation therapy by patients seen in post-COVID clinics. Post-COVID clinic-centered models may have advantages for coordinating referrals to subspecialists and monitoring progress in rehabilitation therapies. Modeling care coordination after these post-COVID clinics or after primary care-centric models used in other chronic diseases may prove to be an efficient way to address the various needs of patients with PCC, especially as the number of patients with long-term sequelae of SARS-CoV-2 infection is anticipated to rise. Additional studies are needed to further investigate the potential impact of different care coordination models, as well as subspecialist and therapist referrals on support for persons with prolonged symptoms.

## Supplementary Information

Below is the link to the electronic supplementary material.

Supplementary Material 1

Supplementary Material 2

## Data Availability

The datasets used and/or analyzed during the current study are available from the corresponding author on reasonable request.

## Abbreviations

PCC: Post-COVID conditions
PCR: polymerase chain reaction
COPD: chronic obstructive pulmonary disease
BMI: body mass index
SARS-COV-2: Severe acute respiratory syndrome coronavirus 2
GI: gastrointestinal
PT/OT: physical therapy/ occupational therapy
PTSD: Post traumatic stress disorder
CT: Computerized tomography
MRI: Magnetic resonance imaging
