# Supplementary material for: Medical complexity and healthcare utilization among patients attending three U.S. post-COVID clinics
Source: BMC Infect Dis. 2025 Sep 26;25:1145. doi: 10.1186/s12879-025-11424-1 (PMC12465321; doi:10.1186/s12879-025-11424-1)
Supplement: Supplementary file 1 — Supplementary Material 1 [file 12879_2025_11424_MOESM1_ESM.docx]

**Supplemental Table 1. Symptoms and Summary Physical Exam Findings ^a^ at Health Care Visits for Adult patients Attending Three Post-COVID Clinics — United States^b^, January 20, 2020–March 31, 2021^c^**

| **Symptoms and Physical Exam Findings** | **Patients with Symptoms or Findings (%)** |
| --- | --- |
| **Symptoms (n=984)** |  |
| Shortness of breath/breathlessness | 583 (59.2) |
| Post-exertional malaise | 449 (45.6) |
| Fatigue | 425 (43.2) |
| Difficulty thinking clearly or concentrating/forgetfulness/memory loss/ brain fog | 421 (42.8) |
| Headache | 369 (37.5) |
| Joint pain or swelling | 322 (32.7) |
| Persistent muscle pain | 316 (32.1) |
| Persistent cough | 309 (31.4) |
| Palpitations (heart racing or pounding) | 293 (29.8) |
| Problems sleeping | 289 (29.4) |
| Dizziness/light headedness | 283 (28.8) |
| Chest pains | 268 (27.2) |
| Weakness in arms or legs / muscle weakness | 227 (23.1) |
| Loss of smell and/ or taste | 191 (19.4) |
| Tingling feeling/ ‘pins and needles’ | 181 (18.4) |
| Feeling sick/vomiting | 153 (15.5) |
| Stomach/abdominal pain | 152 (15.4) |
| Diarrhea | 140 (14.2) |
| Constipation | 119 (12.1) |
| Hair loss (alopecia) | 116 (11.8) |
| Weight loss | 113 (11.5) |
| Skin rash | 111 (11.3) |
| Fever/chills | 99 (10.1) |
| Loss of appetite | 94 (9.6) |
| Swollen ankles | 93 (9.5) |
| Problems with balance | 81 (8.2) |
| Shakiness/tremors | 79 (8.0) |
| Bleeding | 67 (6.8) |
| Problems speaking or communicating | 66 (6.7) |
| Problems passing urine | 61 (6.2) |
| Problems swallowing or chewing | 56 (5.7) |
| Can't fully move or control movement | 33 (3.4) |
| Erectile dysfunction | 29 (2.9) |
| Changes in menstruation | 25 (2.5) |
| Fainting/Blackouts or passing out | 25 (2.5) |
| Can't feel one side of the body or face | 18 (1.8) |
| Lumps or rashes (purple/pink) on toes | 15 (1.5) |
| Seizures | 5 (0.5) |
| **Physical Exam Findings (n=952)** |  |
| No abnormal findings | 546 (57.4) |
| Abnormal findings | 406 (42.6) |
| Missing (Telehealth visits with no physical exam done)  *Specific Abnormal Physical Exam Findings*  Hypoxemia (oxygen < 94%)  Supplemental oxygen at time of visit  Sensory loss  Rash  Hearing loss  Confusion, disorientation, or other abnormal mental status  Tremor/abnormal movements  Gait abnormalities  Hair loss (alopecia, telogen effluvium)  Facial weakness  Dysarthria (slowed or slurred speech)  Ataxia  Aphasia (inability to understand or express speech) | 32 (n/a)  42 (4.3)  34 (3.5)  20 (2.0)  16 (1.6)  16 (1.6)  13 (1.3)  12 (1.2)  7 (0.7)  3 (0.3)  2 (0.2)  2 (0.2)  1 (0.1)  1 (0.1) |

^a^ Counts of patients who had a symptom or physical exam finding at least once during their follow-up.

^b^ Mount Sinai Health System (MSHS), New York City, New York; University of Texas Southwestern Medical Center (UTSW), Dallas, Texas; and CHI Health Creighton University Medical Center (CHI), Omaha, Nebraska.

^c^ Visits occurred ≥ 28 days after the index date through September 30, 2021. The index date is date of PCR or clinical diagnosis for non-hospitalized and date of discharge for hospitalized patients during the acute illness phase of SARS-CoV-2 infection. Diagnosis of SARS-CoV-2-infection occurred between January 20, 2020, to March 31, 2021.
