# Supplementary material for: Medical complexity and healthcare utilization among patients attending three U.S. post-COVID clinics
Source: BMC Infect Dis. 2025 Sep 26;25:1145. doi: 10.1186/s12879-025-11424-1 (PMC12465321; doi:10.1186/s12879-025-11424-1)
Supplement: Supplementary file 2 — Supplementary Material 2 [file 12879_2025_11424_MOESM2_ESM.docx]

**Supplemental Table 2: Laboratory Tests for Adult Patients at Three Post-COVID Clinics —**

**United States^a^, January 20, 2020–March 31, 2021^b^**

| **Test** | **Patients with ≥1 Abnormal Lab Value**  **n (%)** | **Patients with Available Lab Values**  **n** |
| --- | --- | --- |
| Serum glucose | 375 (47.6) | 787 |
| Serum albumin | 246 (31.3) | 787 |
| Estimated glomerular filtration rate | 244 (31.0) | 787 |
| Hemoglobin | 226 (28.7) | 787 |
| C-reactive protein | 204 (26.1) | 783 |
| Hematocrit | 189 (24.0) | 787 |
| Total white blood cell count | 188 (23.9) | 787 |
| Aspartate aminotransferase | 185 (23.5) | 787 |
| Serum potassium | 178 (22.6) | 787 |
| Serum calcium | 177 (22.5) | 787 |
| Serum creatinine | 141 (17.9) | 787 |
| Blood urea nitrogen | 140 (17.8) | 787 |
| Carbon dioxide | 131 (16.6) | 787 |
| Alanine aminotransferase | 128 (16.3) | 787 |
| Serum ferritin | 122 (15.6) | 783 |
| Serum chloride | 120 (15.2) | 787 |
| Low-density-lipoprotein cholesterol | 114 (14.6) | 782 |
| Serum sodium | 112 (14.2) | 787 |
| Hemoglobin a1c | 102 (13.0) | 782 |
| Platelets | 92 (11.7) | 787 |
| High-density-lipoprotein cholesterol | 73 (9.3) | 781 |
| Total protein | 71 (9.0) | 787 |
| Serum alkaline phosphatase | 65 (8.3) | 787 |
| Triglycerides | 61 (7.8) | 781 |
| Pro B natriuretic peptide | 56 (7.2) | 783 |
| Total cholesterol | 56 (7.2) | 782 |
| Total bilirubin | 41 (5.2) | 787 |
| Partial thromboplastin time | 31 (4.0) | 783 |
| International normalized ratio | 27 (3.5) | 782 |
| Troponin I | 24 (3.1) | 785 |
| Serum phosphate | 17 (2.2) | 782 |
| Microalbumin/creatinine ratio | 14 (1.8) | 781 |
| Brain natriuretic peptide | 10 (1.3) | 775 |
| Direct bilirubin | 7 (0.9) | 782 |
| Troponin T | 2 (0.3) | 780 |
| Total T-cell count | 1 (0.1) | 781 |
| CD4/CD8 ratio | 0 (0) | 781 |

^a^ Mount Sinai Health System (MSHS), New York City, New York; University of Texas Southwestern Medical Center (UTSW), Dallas, Texas; and CHI Health Creighton University Medical Center (CHI), Omaha, Nebraska.

^b^ Visits occurred ≥ 28 days after the index date through September 30, 2021. The index date is date of PCR or clinical diagnosis for non-hospitalized and date of discharge for hospitalized patients during the acute illness phase of SARS-CoV-2 infection. Diagnosis of SARS-CoV-2-infection occurred between January 20, 2020, to March 31, 2021.
